# A molecular diffusion based utility model for *Drosophila *larval phototaxis

**DOI:** 10.1186/1742-4682-9-3

**Published:** 2012-02-02

**Authors:** Zhejun Gong, Zhefeng Gong

**Affiliations:** 1 College of Logistics Engineering, Wuhan University of Technology, Wuhan, China, 430073; 2 Institute of Biophysics, Chinese Academy of Sciences, 15 Datun Road, Beijing, China, 100101

**Keywords:** *Drosophila *larva, Phototaxis, Decision making, Utility

## Abstract

**Background:**

Generally, utility based decision making models focus on experimental outcomes. In this paper we propose a utility model based on molecular diffusion to simulate the choice behavior of *Drosophila *larvae exposed to different light conditions.

**Methods:**

In this paper, light/dark choice-based *Drosophila *larval phototaxis is analyzed with our molecular diffusion based model. An ISCEM algorithm is developed to estimate the model parameters.

**Results:**

By applying this behavioral utility model to light intensity and phototaxis data, we show that this model fits the experimental data very well.

**Conclusions:**

Our model provides new insights into decision making mechanisms in general. From an engineering viewpoint, we propose that the model could be applied to a wider range of decision making practices.

## Background

Animals (including human beings) face the problem of choice making at both individual and population levels. *Drosophila *is a model animal that exhibits choice behaviors in various taxis responses. Decision making theories employ the concept of utility as a basis for choice. Utility maximization is a basic presumption of behavioral decisions [1]. If an animal consistently chooses one option in a given set of circumstances, that option is assigned a higher utility than the competing options at the time of decision. Insofar as choice is adaptive, the utilities of goal objects and activities can be considered subjective estimates of potential contributions to fitness [2]. Because utility plays an import role in decision making theories, it is meaningful to study the utility model from animal behaviors to help us make optimal judgments.

Phototaxis is generally considered a form of light dependent preference behavior in animals. In the fruit fly *Drosophila melanogaster*, it is well known that wild type adults show positive phototaxis while negative phototaxis is seen in larvae [3-7]. From time of hatching to the early third instar stage, larvae robustly avoid light [3,8,9]. Immediately prior to pupation, light avoidance declines dramatically and animals become photoneutral, while adult flies are strongly attracted to light [3,8,9]. These behavioral changes undoubtedly reflect an innate search for suitable environments and a quest for survival. Early-instar larvae eat voraciously and, by avoiding light, they immerse themselves in food-rich environments while also avoiding predators. In addition to becoming less photophobic, wandering third instar larvae cease feeding and exit food to pupate. Survival rates are low for larvae that pupate in food. It seems unlikely that a change in visual behavior is the sole reason for larvae to leave the food, but rather that during this stage an array of developmentally programmed behavioral changes occur [9].

Decision making in *Drosophila *larvae exposed to different light conditions can be tested in a simple light/dark choice assay [3,4], in which the larvae are free to move towards their preferred light condition. During the phototaxis assay, larval distribution in the different light conditions changes dynamically as larvae seek the preferred condition. The complete mechanism underlying larval phototaxis is not yet clearly understood, but significant progress has been made in recent years [9]. The two small eyes of the larva are much simpler than the compound eye of the adult. Each larval eye, also termed the 'Bolwig Organ' (BO), is composed of only 12 photoreceptor neurons (PRs), which are divided into two subtypes according to the rhodopsin gene they express. Eight PRs express the green-sensitive rhodopsin6 (rh6) and four express the blue-sensitive rhodopsin5 (rh5) [10-12]. When light is detected by the PRs, signals are sent to downstream 5^th ^lateral neurons (LNs) using acetylcholine (Ach) as the neurotransmitter [3,6,9]. Further downstream, the so-called NP394 neurons are known to control larval light preference, but how these NP394 neurons trigger motor neuron responses at the output layer remains unknown [5].

Diffusion plays a crucial role in brain function because diffusion moves informational substances between cells [13]. To understand how information is processed between cells in larval phototaxis changes, we need to know how mobility and local interactions of molecules lead to variability in light preference. Changes in the extracellular environment are usually transmitted in the cell through changes in the conformation or association of intracellular proteins. In the simplest case, the information contained in the state of these proteins is transmitted through space by their diffusional mobility [14]. That is, on a fundamental level, fluctuations in intracellular or extracellular molecular positions can occur by diffusion [15]. Fick's Second Law, also known as the Diffusion Equation, describes non-steady-state diffusion and is typically used to model molecular mobility [14-16]. Because the Diffusion Equation is nonlinear, the correct parameters can be obtained by global optimization.

In conventional least square (LS) regressions for nonlinear problems, it is not easy to obtain analytical derivatives with respect to target parameters. Even if the derivatives can be obtained analytically or numerically, one must take care to choose the correct initial values for iterative equation-solving processes, because some undesired, locally optimized solutions may also satisfy the equation. Nonlinear problems may possess multiple local minima; finding the global minimum is usually difficult using conventional LS regressions [17]. On the other hand, one can try to match coefficients of the polynomial with least squares fitting by solving a linear system. The linear system is obtained by minimizing the total square error. However, the linear system is ill-conditioned for high polynomial order [18].

The shuffled complex evolution metropolis algorithm (SCEM-UA) is a global-searching algorithm based on improvements of the shuffled complex evolution algorithm (SCE-UA) developed by Duan et al [19]. The SCEM-UA method adopts Markov Chain Monte Carlo theory (MCMC) and uses the Metropolis-Hastings algorithm (MH), replacing the Downhill Simplex method, to obtain a global optimal estimation [19]. The SCEM-UA algorithm is used to estimate mixed Weibull distribution parameters in automotive reliability analysis. The results are compared with maximum likelihood estimation (MLE) results. In published examples, SCEM-UA has been shown to deliver more accurate results than MLE [20]. Although SCEM-UA can successfully obtain the global optimal solution, its performance depends on correct setting of the minimal and maximal limits. In the current study, we improve the SCEM-UA algorithm so that it can optimize the parameter searching space and obtain the optimal solution. This improved algorithm is termed the ISCEM algorithm.

From the above discussion of larval phototaxis neural mechanism, we infer that molecular mobility (of, for example, acetylcholine) plays a critically important role in larval phototaxis processes. In essence, the larvae convert light stimuli to molecular propagation processes. The molecular mobility in larval phototaxis is apparently based on the diffusion of molecules inside or outside neural cells [14-16]. That is, larval photophobia in *Drosophila *is a process of larval molecular movement driven by light intensity. Thus, we can use the molecular diffusion model to describe the larval light avoidance behavior, replacing molecular concentration with light intensity as the driving force. Although the underlying molecular mechanism remains unclear, it is possible that some biological molecules are synthesized at high concentration, and are reduced to lower concentration by diffusion; for example, the neurotransmitters or other neuropeptides involved in photophobic behavior. Based on such understanding, we use the Diffusion Equation as our decision making model and then test its compatibility with the experimental data.

In summary, we propose a utility model derived from molecular diffusion to quantitatively investigate the relationship between light intensity and *Drosophila *larval photophobia, with the aid of a math ISCEM algorithm. By testing the model with experimental data, we find that the dynamic process of larval phototaxis and light intensity-photophobia is well simulated. Although the neural mechanism underlying this utility model is unclear, this model enhances our understanding of decision making mechanisms from an engineering viewpoint. We expect that this model can provide insights into the neural basis of decision making activities.

## Materials and methods

### Fly stock

Fly strain *w^1118 ^*larvae were reared on standard medium [21] under conditions of normal light/dark (LD) cycles. In all experiments, early to mid-3^rd^-instar larvae (72-96h after egg laying) were used.

### Behavioral assay

**Figure 1:**
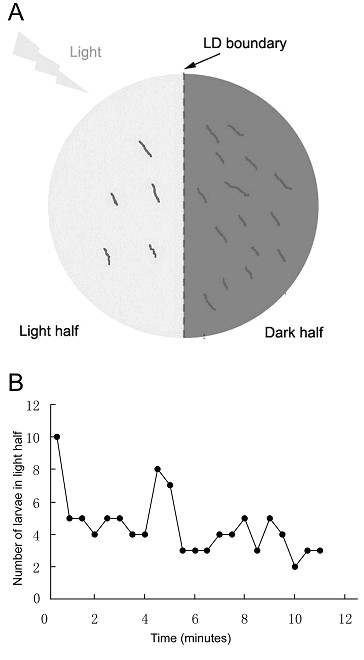
**Larval phototaxis test**. (A) Schematic representation of the testing plate. The light and dark halves of the testing plate are colored light and dark gray, respectively. See Materials and methods for details of the 11-min phototaxis test. (B) Dynamics of *w^1118 ^*3rd-instar larval distribution in the light and dark halves of the testing plate during the 11-min phototaxis test. Twenty larvae were placed on the light side of the LD boundary at the beginning of the test. The number of larvae in the light half was counted every 0.5 min. The testing time of 11 min was used to demonstrate the final larval distribution. Light intensity was 550 lux.

All behavioral tests were performed at room temperature (22-24°C) between 10:00 am and 5:00 pm. The 11-min phototaxis tests were performed following the protocol introduced by Mazzoni et al. with modifications [3,4]. In brief, 8 cm petri dishes containing 1.5% Bacto Agar, with one half of the lid covered with black electrical tape, were illuminated from above using an 11W energy-saving fluorescent light (Leike Inc). Early third instar larvae were removed from food and washed with fresh distilled water. For each test, 20 larvae were placed on the agar surface and allowed to move freely for 11 minutes before their numbers on each side of the testing plate were counted (Figure 1). The light avoidance index (AI) was calculated as AI = (number of larvae in the dark half - number of larvae in the light half)/(number of larvae in the dark half + number of larvae in the light half). Specifically for the larval dynamic distribution analysis, all larvae were initially placed in the light half but at distances of no more than 1 cm from the dark/light boundary. Light intensities were 150, 350, 550, 750 and 950 lux. The corresponding avoidance indices of *w^1118 ^*under these light conditions are shown in Table 1.

**Table 1:** *w*^1118 ^larvae light avoidance indices at different light intensities (Experimental data)

Light intensity	150lux	350lux	550lux	750lux	950lux
AI	0.2	0.4	0.7	0.8	0.9

### Building the utility model

According to Fick's second law of diffusion [16], the spatial distribution of particles emitted from a source evolves as:

(1) $$\partial V/\partial t=D*\partial^{2}V/\partial x^{2}$$

in which *t *is the time, *x *is the distance from the molecule-producing source; *D *is the diffusion coefficient, and *V *is the concentration of the molecule at distance *x *from the source.

Under initial conditions of *t *= 0 and *x *> 0, *V *= 0; under marginal conditions of *t *> 0 and *x *= 0, *V *= *V_s_*. When *t *> 0 and *x *= ∞, *V *= 0.

The solution of equation (1) is

(2) $$V\left(x,t\right)=V_{s}\left[1-erf\left(x/2\sqrt{Dt}\right)\right]$$

where $erf\left(c\right)=\left(2/\sqrt{\pi }\right)\int_{0}^{c}\exp\left(-c^{2}\right)dc$.

From Equation (2), it is apparent that

(3) $$V_{i}\left(t\right)=V_{s}\left[1-erf\left(x/2\sqrt{Dt}\right)\right]$$

where *V_i _*is the output concentration of source *i*.

We emphasize that *Drosophila *larval photophobia is based on molecular movement in the larva, driven by light intensity. As mentioned in the Background section, we understand that larval light avoidance behavior mimics molecular diffusion, and that diffusive processes are involved in photophobia at the cellular level. Equation (3) therefore forms the basis of our decision making model.

### ISCEM algorithm: An improved SCEM-UA algorithm

Suppose *ŷ = η (ζ | θ)*, where *ŷ *× 1 vector of model predictions, *ζ *is an *N *× *n *matrix of input variables and *θ *is a vector of *n *unknown parameters. The SCEM-UA algorithm is given below:

(1) To initialize the process, choose the population size *s *and the number of complexes *q*. The algorithm tentatively assumes that the number of sequences is identical to the number of complexes.

(2) Generate *s *samples from the prior distribution {*θ_1 _,θ_2_*,...,*θ_s_*} and compute the posterior density {*p*(*θ*^(1) ^| **y**),*p*(*θ*^(2) ^| **y)**,...,*p*(*θ*^(s) ^| **y)**} at each point [19].

(3) Sort the points in order of decreasing posterior density and store them in an array D[1:*s*,1:*n*+1], where *n *is the number of parameters, so that the first row of D represents the point with the highest posterior density. The extra column stores the posterior density. Initialize the starting points of the parallel sequences, S^1^,S^2^,...,S*^q^*, such that S*^k ^*is D[*k*,1:n+1], where *k *= 1,2,...,*q*.

(4) Partition D into *q *complexes C^l^,C^2^,...,C*^q^*, each containing *m *points, such that the first complex contains every *q(j - 1) *+ 1 ranked point, the second complex contains every *q(j - 1) *+ 2 ranked point of D, and so on, where *j *= 1,2,...,*m*.

(5) Initialize L,T,AR_min_, c_n_. For each C*^k^*, call the SEM algorithm [19] and run it L times;

(6) Unpack all complexes C back into D and rank the points in order of decreasing posterior density.

(7) Check Gelman and Rubin (GR) convergence statistic. If convergence criteria are satisfied, stop; otherwise, return to step 4.

The ISCEM algorithm is given below:

(1)Suppose I_min_≤*θ*≤I_max _, I_min _and I_max _are interval vectors of *θ*. The initial I_max _is set to be very large. Run the SCEM-UA algorithm and let the output parameter vector with highest posterior density (*p_o_*) be *θ_o_*. Set I_max _= *θ_o_*.

(2)Run the SCEM-UA algorithm again, and let the output parameter vector with highest posterior density (*p_w_*) be *θ_w_*. If || *p_o _*- *p_w _*|| ≤ *ε *, where *ε *> 0, go to step (4); otherwise set *θ_o _*= *θ_w_*.

(3) If *p_o _*≤ *p_w _*, let I_max _= *θ_w _*; otherwise, let I_min _= *θ_w _*. Let *p_o _*= *p_w_*, go to step (2).

(4) Output *θ_w _*.

## Results

Simulating *Drosophila *larval phototaxis dynamics with the model

To use our utility model to simulate the relationship between light intensity and *Drosophila *larval photophobia, Equation (3) is rewritten as

(4) $$f\left(t\right)=\alpha *l*\left[1-erf\left(\beta /\sqrt{t}\right)\right]$$

in which *f*(*t*) denotes photophobia (assessed by AI), *α *and *β *are constants, *l *is light intensity, and *t *is the time of light exposure (in minutes).

When the light intensity is large enough and/or the testing time is long enough, the larvae may all crawl to the dark section of the plate (i.e. AI = 1). Under these circumstances, the larvae obtain no stimulus from the light. To account for this phenomenon, if *f*(*t*) calculated from equation (4) exceeds 1, its value is set to 1; that is, an upper bound of 1 is imposed on *f*(*t*).

**Figure 2:**
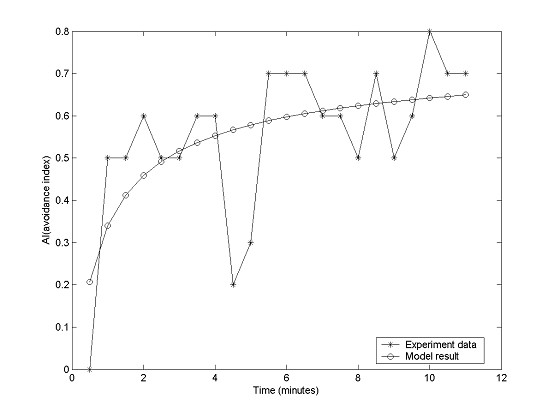
**Comparison between experimental data and model result (at 550 lux)**. Experimental data are represented by asterisks, AI predictions (output by the model) by circles.

To validate the model, we simulate experimental data. Best estimates of the model parameters are obtained using the ISCEM method, which can realize parameter estimation of complex functions and has a global optimal search capability. Experimental data shown in Figure 2 are used as inputs.

Feeding these data into the ISCEM algorithm, the parameters of the model are

*α *= 0.001459, *β *= 0.56532

and the model equation becomes

(5) $$f=0.001459*550*\left[1-erf\left(0.56532/\sqrt{t}\right)\right]$$

With the form of the model now specified, we then predict the time course of the AI under light intensity 550 lux. The predicted data are compatible with experimental AI records, as shown in Figure 2.

The comparison statistics between model prediction and experimental data are: mean of error = -0.002, standard deviation of error = 0.14, mean of absolute error = 0.11, standard deviation of absolute error = 0.09. The determination coefficient R^2 ^= 0.42. The F-value is 14.28 and F_0.01_(1,20) is 8.10. Because the F-value > F_0.01_(1,20), the model passes the F-test. Considering that biological data are inherently prone to experimental noise, the model provides good matches to the experimental data.

### Validation of the model with experiment data from other light intensities

We externally validate the model further by investigating the relationship between AI and light intensity. In estimates of external validity, some samples should be excluded from the parameter estimation [22]. We replace 550 lux with varying light intensity *l*. The model described by Equation (5) now becomes:

(6) $$f=0.001459*l*\left[1-erf\left(0.56532/\sqrt{t}\right)\right]$$

**Figure 3:**
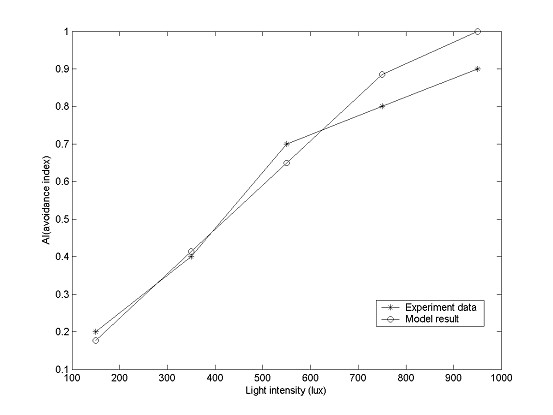
**Comparison between AI value from Table 1 (experimental) and Table 2 (predicted)**. Experimental data are represented by asterisks, AI predictions (output by the model) by circles.

Setting *t *= 11, we can compute the AI data for different light intensities (See Table 2). The predicted data align well with experimental AI records, as shown in Figure3. The comparison statistics between model prediction and experimental data are: mean of error = -0.03, standard deviation of error = 0.07, mean of absolute error = 0.05, standard deviation of absolute error = 0.04. The determination coefficient is 0.94. Given these statistics, we conclude that the data predicted from the model closely match the experimental data.

**Table 2:** *w*^1118 ^larvae light avoidance indices at different light intensities (Model prediction)

Light intensity	150lux	350lux	550lux	750lux	950lux
AI(Model result)	0.18	0.41	0.65	0.89	1.0(1.12)

(Note: When light intensity is 950lux, the model predicts an AI of 1.12. Because this exceeds the imposed upper bound of 1, it is set equal to 1.)

## Conclusions

In this paper, we build a utility model to simulate the light preference in *Drosophila *larval phototaxis behavior. The model can successfully simulate both the dynamics of larval redistribution and the relationship between avoidance index and light intensity, suggesting that our model can be developed into a new form of decision making model.

Fick's second law of diffusion, the basis of the model, has been widely applied in engineering and material studies in addition to biological/medical studies [23-27]. The molecular diffusion process, which decreases the molecular concentration at the source by spreading the particles through a wider volume, is mimicked in certain animal behaviors. When the molecular concentration in the diffusion equation is replaced with the outside stimulus intensity, this model can simulate the processes of various fly behavior preferences, such as phototaxis, thermotaxis, chemotaxis, odortaxis, etc. [5,28-30].

As the molecular diffusion based utility model can correctly simulate the experimental data of larval phototaxis, it is natural to postulate that specific molecules diffuse around and along the neural network to generate the phototaxis behavior. Currently, how this biological mechanism functions is poorly understood. It is known that neuropeptides and neurotransmitters (such as acetylcholine that mediates signaling between photoreceptors and secondary neurons) play key roles in larval phototaxis [6,9]. Further study on related neurotransmitters and signaling neuropeptides is required to verify this model at the molecular level.

Since molecular mobility is the neural basis of animal behavior, it is reasonable to postulate that all animal physiological and behavioral functions can be simulated with such a model. For the larval photophobia investigated in this paper, the experimental data matches well with model prediction. We anticipate that this utility model may be applied to decision making behavior in humans, which is very similar to animal choice behavior [31], though more experimental data are needed to confirm this. In any case, the model may aid our understanding of the human decision making process. With further optimization and refinement, the model could provide a new tool by which to study generic decision making behaviors. To this end, the model must be tested over a wide range of choice behaviors; this goal will be addressed in future studies.

## Competing interests

The authors declare that they have no competing interests.

## Authors' contributions

ZG (Zhejun Gong) conceived the idea and wrote the manuscript. ZG (Zhefeng Gong) designed and undertook the experiments. All authors read and approved the final manuscript.
